# Crosstalk between cyclic-di-guanosine monophosphate and the sensor kinase MtrB regulates MtrA-dependent genes, bacterial growth, biofilm formation and lysosomal trafficking of Mycobacterium tuberculosis

**DOI:** 10.1099/mic.0.001532

**Published:** 2025-02-07

**Authors:** Shreya Bagchi, Arun Kumar Sharma, Soumya Mal, Manikuntala Kundu, Joyoti Basu

**Affiliations:** 1 Department of Chemical Sciences, Bose Institute, 93/1 APC Road, Kolkata 700009, India; 2 Department of Biological Sciences, Bose Institute, Unified Academic Campus, EN 80, Sector V, Bidhan Nagar, Kolkata 700091, India

**Keywords:** biofilm, c-di-GMP, lysosomal trafficking in macrophages, *Mycobacterium tuberculosis*, sensor kinase MtrB

## Abstract

Cyclic-di-guanosine monophosphate (c-di-GMP) plays an important role in bacterial signalling networks. C-di-GMP exerts a regulatory function through binding to diverse molecules that include transcription factors, riboswitches and sensor kinases (SKs), thereby regulating diverse processes. Here, we demonstrate the crosstalk between c-di-GMP and the SK MtrB of *Mycobacterium tuberculosis*. MtrB phosphorylates and regulates its cognate response regulator MtrA. C-di-GMP binds directly to the cytosolic domain of MtrB to inhibit its autophosphorylation. C-di-GMP levels in *M. tuberculosis* were manipulated by overexpressing a c-di-GMP synthesizing enzyme *ydeH* and a degrading enzyme *rv1357c*. We demonstrate that overexpression of *ydeH* lowers growth of the bacterium both *in vitro* and in *M. tuberculosis* grown in macrophages. This is in conformity with lowered expression of *mtrA* and selected genes of the *mtrA* regulon involved in cell wall turnover in the *ydeH*-overexpressing strain compared to the parent strain. We also demonstrate that overexpression of *ydeH* in *M. tuberculosis* hinders biofilm formation, whereas overexpression of *rv1357c* has the opposite effect. Neither of the two genes could rescue the biofilm defective phenotype of the MtrB knock out mutant (*ΔmtrB*), suggesting that c-di-GMP exerts its role on biofilm formation through MtrB. Finally, we show by fluorescence microscopy that the trafficking of *M. tuberculosis* overexpressing *ydeH* is significantly higher than that of the parent strain and that this is linked to reduced expression of the MtrB-dependent genes *esxG* and *esxH*, which play a role in subversion of lysosomal trafficking of *M. tuberculosis*. These results provide important new insight into the crosstalk between c-di-GMP and MtrB in *M. tuberculosis*.

## Introduction

The World Health Organization’s Global Tuberculosis Report for 2023 estimates that a total of 10.6 million people fell ill with tuberculosis (TB) in 2022, with most TB cases reported in South-East Asia, Africa and the Western Pacific [1]. TB incidence is estimated to have increased by 1.9% between 2020–2021 and 2021–2022. Globally in 2022, there were an estimated 1.13 million deaths among HIV-negative people and an estimated 167 000 deaths among people with HIV. Considering that there was a reversal in the trend of declining TB incidence between 2020 and 2022 due to the emergence of the SARC-CoV-2 pandemic, it is necessary to strengthen therapeutic management of TB to contain and limit its spread. Resistance to rifampicin, isoniazid and a host of second-line TB drugs makes it necessary to identify alternate targets that are crucial for the establishment of infection by *Mycobacterium tuberculosis*, the causative agent of TB. One of the means towards this end is to better understand the signalling systems that enable the bacterium to sense and respond appropriately to the host environment. Two-component systems (TCSs) comprising a sensor kinase (SK) and its cognate response regulator (RR) enable the bacterium to sense and respond to various stresses such as hypoxia, acidic pH, starvation and ionic imbalances [2,3]. Signalling by TCSs is triggered when the SK senses stress and is phosphorylated. This triggers a phospho relay in which the phosphate on the SK is transferred to an aspartate on the RR. The phosphorylated RR binds to specific DNA sequences on its targets and regulates transcription. The focus of the present study is the SK MtrB, a component of the TCS MtrAB, and its interaction with the cyclic-di-guanosine monophosphate (c-di-GMP) signalling pathway.

MtrAB is the likely counterpart of the YycFG (or WalKR) TCS of the low G+C Gram-positive bacteria, such as *Bacillus subtilis*, *Staphylococcus aureus*, *Streptococcus pneumoniae*, *Streptococcus pyogenes*, *Listeria monocytogenes* and *Enterococcus faecalis* [4,6]. In *Corynebacterium glutamicum*, deletion of MtrAB impacts cell morphology, antibiotic susceptibility and osmoprotection [7].

MtrB can be knocked out in *M. tuberculosis* [8], whereas MtrA is essential. Phosphorylated MtrA binds, among other targets, to the origin of replication (*oriC*), *fbpB*, *ripA*, *rpfA*, *rpfB* and *dnaA* to regulate both replication and cell wall synthesis [9,13]. The depletion of *mtrA* in *Mycobacterium smegmatis* is associated with filamentation and cells with branches and bulges [11]. MtrB regulates the expression of the MtrA targets *ripA*, *fbpB* and *ftsI* [14]. It regulates cell morphology, cellular morphology, biofilm formation and survival of *M. tuberculosis in vivo* [8]. MtrB localizes to cell septa and interacts with FtsI to regulate septal localization of FtsI [11]. The sequestration of autophosphorylated SKs by non-cognate RRs has been postulated to preclude an amplified response to a weak stimulus [15]. In the unphosphorylated state, the binding affinity of MtrB for MtrA is lower than that of phosphorylated MtrB [15]. *M. tuberculosis* uses multiple cyclic nucleotides, including cyclic adenosine monophosphate, cyclic-di-adenosine monophosphate (c-di-AMP) and c-di-GMP, to regulate its response to external signals and also to regulate host cell signalling [16]. C-di-GMP regulates motility, adherence, biofilm formation, virulence and cell cycle progression in various bacterial species [17,18]. In *Caulobacter crescentus*, binding of c-di-GMP to the kinase CckA shifts it into phosphatase mode to allow chromosome replication [19]. C-di-GMP coordinates cell cycle and morphogenesis in *C. crescentus* [20]. C-di-GMP binds to the SK RavS to control RavS-RavR phosphotransfer and bacterial lifestyle transition between virulence and swimming [21]. C-di-GMP controls metabolic activity in *Pseudomonas aeruginosa* [22].

In *M. smegmatis*, c-di-GMP acts as a signalling molecule that regulates expression of transcription factors such as HpoR to regulate the antioxidant defence system of *M. smegmatis* [23]. C-di-GMP also regulates keto mycolic acid synthesis and biofilm formation in an Lsr2 (a nucleoid-associated protein) dependent manner [24]. In *Mycobacterium bovis* Bacillus Calmette Guerin (BCG), direct interaction between ArgR and c-di-GMP regulates nitrate metabolism during adaptation of the bacterium to hypoxia. As c-di-GMP accumulates during hypoxia, it blocks the inhibitory activity of ArgR on the arginine metabolism gene cluster *argC-H* [25]. C-di-GMP phosphodiesterase gene deletion in *M. bovis* BCG leads to transcriptome remodelling, which includes genes of the MtrA, ArgR and DosR regulons [26]. C-di-GMP influences TCS signalling in mycobacteria. The SK PdtaS of mycobacteria binds c-di-GMP to regulate signalling mediated by the PdtaS-PdtaR TCS [27].

The signal(s) responsible for the regulation of MtrB is not known. Here we have tested the hypothesis that c-di-GMP regulates a number of processes in *M. tuberculosis* by virtue of its ability to regulate the SK MtrB. We identify MtrB as a novel binding partner of c-di-GMP. C-di-GMP binds to the cytosolic domain of MtrB and attenuates MtrB autophosphorylation. Using genetically manipulated strains expressing higher or lower levels of c-di-GMP compared to the wild-type, we demonstrate that elevated levels of c-di-GMP lead to repressed levels of expression of genes of the *mtrA* regulon. This is associated with changes in mycobacterial growth, biofilm formation, survival in macrophages and trafficking of *M. tuberculosis* to lysosomes. These results shed light on the crosstalk between c-di-GMP and the SK MtrB and its downstream effects.

## Methods

### Bacterial strains and growth conditions

*Escherichia coli* strains Top10 and DH5α were used for cloning, while *E. coli* Origami (DE3), BL21 (DE3) and C41 (DE3) were used for recombinant protein expression. *E. coli* was grown in Luria−Bertani (LB) Miller broth with shaking at 150 r.p.m. or on LB agar (Becton Dickinson, Difco) at 37 °C. *M. tuberculosis* H37Rv and other genetically manipulated *M. tuberculosis* strains were cultured in Middlebrook (MB) 7H9 broth (Difco) supplemented with 10% v/v albumin-dextrose-catalase (ADC) (Difco or Hi-Media Laboratories, India), 0.05% Tween 80 (Hi-Media Laboratories, India) and appropriate antibiotics where required, at 37 °C with shaking at 120 r.p.m. For solid media, MB 7H11 agar (Difco), supplemented with 10% v/v oleic acid albumin dextrose catalase (OADC) and antibiotics as necessary, was used for plating *M. tuberculosis* strains. For *E. coli*, kanamycin sulphate (Sigma), ampicillin (Sigma) and hygromycin (Invitrogen) were used at concentrations of 50, 100 and 200 µg ml^−1^, respectively. For *M. tuberculosis*, kanamycin and hygromycin were used at concentrations of 20 and 50 µg ml^−1^, respectively. A list of strains and plasmids has been provided in Table 1. Colony-forming units (CFUs) were assessed by growing *M. tuberculosis* and its derivative strains to mid-log phase, diluting to an optical density of 0.1 at 600 nm (OD_600_) and plotting CFUs of cultures grown for different periods of time.

**Table 1.** List of bacterial strains and plasmids used in this study

Strain	Description	Reference or source
*E. coli* Top10	*F^-^mcrAΔ (mrr–hsdRMS–mcrBC) ψ80lacZ Δ M15 Δ lacX74 nupG recA1 araΔ139D(ara–leu) 7697 galE15 galK16 rpsL(Str^R^) endA1 λ^-^*	Invitrogen, USA
*E. coli* DH5α	*recA1, endA1, gyrA96, thi; relA1,hsdR17(rK^-^, mK^+^), supE44, φ80∆lacZ∆M15, ∆lacZ(YA-argF)UE169*	Stratagene, USA
*E. coli* BL21 (DE3)	*F^-^ompT gal [dcm][lon] hsdS_B_ (r_B_^-^m_B_^-^; an E.coli B strain*) with *DE3,* aλ prophage carrying the *T7 polymerase* gene	Novagen, USA
*E. coli* C41 (DE3)	*F^–^ompThsdSB (r_B_^-^m_B_^-^) gal dcm (DE3),* a λ prophage carrying the *T7 polymerase* gene	Lucigen, USA
Origami B (DE3)	*F ^–^ompThsdSB(rB ^-^mB^-^) gal dcm lacY1 ahpC (DE3) gor522:: Tn10trxB (Kan^R^,Tet^R^*)	Gift from Dr. Deepak Kumar Saini (IISc, Bangalore)
*M. tuberculosis (Mtb*) H37Rv	Wild-type	IICGEB, India
*M. tuberculosis*H37RV: *∆mtrB*	*mtrB* deletion mutant of *M. tuberculosis* H37RV	[8]
*Mtb-pMV261*	*Mtb*, carrying pMV261, *hyg^R^*	This study
*Mtb-rv1357c-*pMV261	*Mtb* carrying *rv1357c* with C-terminal-his fusion in pMV261, *hyg^R^*	This study
*Mtb-ydeH-*pMV261	*Mtb* carrying *ydeH* with C-terminal-his fusion inpMV261, *hyg^R^*	This study
*∆mtrB-pMV261*	*mtrB deletion mutant of H37Rv*, carrying pMV261, *kan^R^* and *hyg*^R^	This study
*∆mtrB-rv1357c-*pMV261	*mtrB deletion mutant of H37Rv*, carrying *rv1357* with C-terminal-his fusion in pMV26, *kan^R^* and *hyg^R^*	This study
*∆mtrB-ydeH-*pMV261	*mtrB deletion mutant of H37Rv*, carrying *ydeH* with C-terminal-his fusion in pMV26	This study
MtrBpProEx-HTB	*ori ColE1* pBR322 origin, f1-origin, *Ptrc*, *lacI*,*Amp*^R^, N-terminal 6X His translationalfusion, protein expression vector	Gift from Dr. Deepak Kumar Saini (IISc, Bangalore)
pMV261Hyg^R^	pBR322 origin, *oriM*, *hyg^R^*	This study

### Expression and purification of recombinant proteins

The recombinant His-tagged construct of the cytoplasmic domain of MtrB (hereafter referred to as MtrB) was a gift from Dr. Deepak Saini at the Indian Institute of Science in Bengaluru, India. MtrB was expressed in *E. coli* Origami (DE3) and induced with 500 µM of isopropyl *β*-d-1-thiogalactopyranoside. Cells were harvested and lysed in lysis buffer containing a protease inhibitor cocktail (Cell Signalling Technology), 2 mg ml^−1^ lysozyme, 0.2% (v/v) Triton X-100 and 5 µg ml^−1^ DNase-I, using freeze/thaw cycles. His-tagged MtrB was purified from cell-free supernatants using Ni^2+^-NTA agarose chromatography [8]. Briefly, after binding of the lysate to Ni^2+^-NTA agarose, the column was washed sequentially with equilibration buffer (50 mM sodium phosphate, pH 7.5, supplemented with 5% glycerol and 100 mM NaCl) containing 10 and 20 mM of imidazole, respectively. His-tagged protein was eluted using equilibration buffer containing 250 mM of imidazole. Purified fractions of the recombinant protein were dialysed against imidazole-free dialysis buffer, concentrated using a Sartorius 10 kDa molecular weight cut-off (MWCO) membrane filter and stored in the presence of 10% (v/v) glycerol at −80 °C. MtrA expression and purification were performed as described by Sharma *et al*. [13]. The cytosolic domain of STING (hereafter referred to as STING) was purified as a His-tagged protein as described by Sharma *et al*. [28].

### UV cross-linking assay of binding of biotinylated cyclic di-nucleotides

Binding of c-di-GMP to MtrB was assayed as described earlier [29]. The cytosolic domain of MtrB at different concentrations was incubated with biotinylated cyclic di-nucleotides (c-di-nucleotides) [2′-biotin-AHC-c-di-GMP or 2′-biotin-AHC-c-di-AMP (BioLog Inc., Hayward, CA, USA)] (10 µM) in a total volume of 10 µl in 50 mM Tris/HCl (pH 8.0) containing 100 mM NaCl at 4 °C with gentle shaking for 5 min. The mixture was subjected to cross-linking by exposure to UV irradiation at a setting of 1500 (×100 µjoules cm^−2^) on ice for 4 min. Cross-linking was stopped by adding 2.5 µl of 5× protein denaturing dye followed by heating in a dry bath for 5 min. Denatured, cross-linked samples were resolved on 12.5% SDS-polyacrylamide gels and transferred to polyvinylidene fluoride (PVDF) membranes. For blocking, membranes were incubated with 5% non-fat dry milk (NFDM) dissolved in 1× Tris-buffered saline (1× TBS) at room temperature for 1 h. After washing three times with 1× TBS containing 0.05% Tween 20 (1× TBST), membranes were incubated at room temperature for 1 h with horseradish peroxidase (HRP) conjugated streptavidin antibody (Millipore, Cat. No. 18152, diluted 1:500, in 1× TBST) and reprobed with MtrB antibody. Blots were developed using a chemiluminescence detection reagent (Cell Signalling Technology).

### *In vitro* kinase assay

The autokinase assay was adapted from Agrawal *et al*. [30]. Briefly, purified MtrB was incubated at room temperature in kinase buffer (50 mM Tris/HCl, pH 8.0, 50 mM KCl and 10 mM MgCl_2_) containing 50 µM ATP and 1 µCi of [*γ*^−32P^] ATP (>3500 Ci mmol^−1^, BRIT) for different periods of time. Reactions were terminated by adding sodium dodecyl sulphate–polyacrylamide gel electrophoresis (SDS/PAGE) gel loading buffer. Samples were separated by 12.5% SDS/PAGE and scanned using a Typhoon Trio plus imaging system (GE Healthcare). Where necessary, MtrB autokinasing was carried out in the absence or in the presence of c-di-GMP (Sigma-SML1228). The reactions were stopped after 40 min and proteins were separated on 12.5% SDS-polyacrylamide gels. For MtrA transphosphorylation, phosphorylated MtrB was incubated with MtrA in kinase buffer for 10 min at room temperature in the absence or in the presence of c-di-GMP, followed by separation by SDS/PAGE and visualization using the Typhoon Trio Plus Imager.

### Phosphatase assay

In order to determine the phosphatase activity of MtrB using phospho-MtrA as substrate, MtrA was phosphorylated using the histidine kinase EnvZ from *E. coli* as described [13]. A recombinant MalE–EnvZ construct (a gift from Dr. M. Igo, University of California, Davis, CA, USA) was transformed into *E. coli* BL21 (DE3). EnvZ was expressed, induced and lysed as described previously by Sharma *et al*. [13]. The EnvZ-expressing lysate was incubated with amylose resin (NEB) in amylose resin binding buffer (20 mM Tris/HCl, pH 7.4, 1 mM EDTA, 200 mM NaCl) for 30 min at 4 °C on a rotator. The resin was washed using wash buffer to remove traces of unbound EnvZ. Finally, resin-bound EnvZ was autophosphorylated with [*γ*^-32P^] ATP in kinase buffer containing 50 µM ATP for 30 min at 37 °C. The resin was washed with kinase buffer to remove excess [*γ*^-32P^] ATP. MtrA was phosphorylated with resin-bound EnvZ~P in kinase buffer for 15 min at room temperature. MtrA~P was carefully removed and filtered through a 10 kDa MWCO filter device (Vivaspin, Sartorius) to remove excess ATP and MgCl_2_. Non-phosphorylated MtrB was then added to the purified MtrA~P in phosphatase buffer (50 mM Tris/HCl, pH 8.0, 50 mM KCl, 5 mM MgCl_2_) in the presence or absence of c-di-GMP. The mixture was incubated at room temperature. Aliquots of 10 µl were removed from the reaction mixture at different time points and denatured in 1× SDS/PAGE loading dye. The samples were resolved by SDS/PAGE and analysed by phosphoimaging using the Typhoon Trio Plus Imager.

### Cloning of *rv1357c* and *ydeH* for mycobacterial expression, preparation of cell lysates and Western blotting

For overexpression of *rv1357* or *ydeH* in *M. tuberculosis* (wild-type or *ΔmtrB*), each gene carrying a hexa-histidine tag at the C-terminus was cloned in pMV261. Briefly, for *rv1357c* overexpression, the gene was PCR amplified from *M. tuberculosis* genomic DNA (BEI Resources, NIAID, NIH, USA) using a specific primer pair (Table 2) and cloned into pMV261 (an *E. coli*-mycobacteria shuttle vector carrying the constitutive *hsp60* promoter) between the BamHI and ClaI sites with an in-frame C-terminal His-tag. Similarly, the *ydeH* gene was amplified from an *E. coli* K12 (NEB-E4104) using a specific primer pair (Table 2) and cloned as described above. Constructs were then electroporated into *M. tuberculosis* H37Rv or *ΔmtrB* to obtain *M. tuberculosis* overexpressing *rv1357c* (*Mtb*-pMV261-*rv1357c*), *ΔmtrB* overexpressing *rv1357c* (*ΔmtrB*-pMV261-*rv1357c*), or *M. tuberculosis* overexpressing *ydeH* (*Mtb*-pMV261-*ydeH*) and *ΔmtrB* overexpressing *ydeH* (*ΔmtrB*-pMV261-*ydeH*), which were selected for growth on plates containing kanamycin or kanamycin and hygromycin. Cultures were grown in MB 7H9 broth supplemented with 10% ADC, 0.05% Tween 80 and appropriate antibiotics, up to an OD_600_ of 0.6. Cells were harvested, resuspended in lysis buffer [phosphate-buffered saline (PBS) containing protease inhibitor cocktail] with 100 micron glass beads, lysed in a Mini bead beater, centrifuged at 16 000 *g* in a microfuge at 4 °C for 30 min, and the cell-free lysate was recovered. Lysates were analysed by SDS/PAGE followed by transfer to PVDF membranes using a semi-dry transfer apparatus (Bio-Rad). Membranes were incubated in 5% NFDM for 1 h at room temperature in 1× TBST (TBS containing 0.05% Tween 20), washed with TBST, incubated overnight at 4 °C and probed with anti-His antibody (Abcam). After washing, the membranes were incubated in 5% NFDM and immunoblotted with HRP-conjugated goat anti-rabbit (or anti-mouse) IgG (Advansta). The membranes were washed with TBST, followed by development with a chemiluminescence detection reagent (Cell Signalling Technology). Reprobing was done with GroEL2 antibody (BEI Resources, NIAID, NIH, USA).

**Table 2.** List of primers used for cloning

Sl. no.	Primer sequence 5′ to 3′	Description
P1	ATT**GGATCC**AATGGATCGTTGTTGTCAG	Forward primer for cloning *rv1357c* with C-terminal *his* tag fusion in pMV261
P2	ATTGGTACC**ATCGAT**TCAGTGGTGGTGGTGGTGGTG	Reverse primer for cloning *rv1357c* with C-terminal *his* tag fusion in pMV261
P3	TT**GGATCC**TATGATCAAGAAGACAACG	Forward primer for cloning *ydeH* with C-terminal *his* tag fusion in pMV261
P4	ATT**AAGCTT**AACTCGGTTAATCACATT	Reverse primer for cloning *ydeH* with C-terminal *his* tag fusion in pMV261

Restriction sites are indicated in bold letters.

### Determination of bacterial c-di-GMP levels by HPLC

The c-di-GMP levels of bacterial cells were measured by high-performance liquid chromatography (HPLC) [31]. *M. tuberculosis* strains were grown to OD_600_≈1.0 in MB 7H9 with 10% OADC and harvested by centrifugation. The bacteria (250 mg dry weight) were resuspended in extraction buffer containing acetonitrile:methanol:water (2:2:1). The suspension was incubated on ice for 15 min, followed by boiling at 95 °C for 10 min and then cooled down on ice. The lysates were centrifuged for 10 min at 4 °C at 20 800×*g* and then transferred into fresh tubes. The extraction was repeated twice. The pooled extracts were vacuum dried and dissolved in sterile water. Next, 10 µl of each sample was injected and separated by reverse-phase HPLC using a Shimadzu HPLC on a C-18 column (4.6 mm×150 mm, Waters Spherisorb) with eluent A (10 mM ammonium acetate and 0.1% acetic acid) and eluent B (methanol) in binary gradient mode. The sample flow rate was 0.6 ml min^−1^ with a column temperature of 30 °C. Nucleotides were monitored at 254 nm. Standard c-di-GMP (Sigma-SML1228) was run to identify the c-di-GMP peak. A standard curve was generated by injecting different concentrations of c-di-GMP (0.25–1 mM) and plotting peak areas vs. c-di-GMP concentrations. Concentrations of c-di-GMP in each sample were calculated from the individual peak areas.

### Biofilm formation

Log-phase cultures of *M. tuberculosis* strains were diluted 1:100 in 5 ml of Sauton’s medium without detergent in T25 non-vented tissue culture flasks. The caps were wrapped with parafilm, and flasks were incubated at 37 °C for 6 weeks. Caps were loosened after 4 weeks of incubation to facilitate biofilm growth. After 6 weeks, media were aspirated with a Pasteur pipette, and the film was allowed to dry. Crystal violet (CV) was added to the films and washed with water, followed by reconstitution in alcohol. Absorbance at 595 nm was measured to quantify the stain associated with each sample. Viability testing in biofilms was performed as described [32]. Briefly, biofilms were allowed to form in 48-well tissue culture plates. Biofilm encased cells were released and resuspended in MB 7H9+0.05% Tween 80 at 37 °C for 6 h with shaking, followed by serial dilution and plating on MB 7H11 agar plates supplemented with 10% OADC, incubation at 37 °C for 5 weeks and enumeration of CFUs.

### Cell culture and infections

The murine macrophage cell line RAW264.7 was obtained from the National Centre for Cell Science, Pune, India. RAW264.7 cells were maintained in RPMI 1640 medium (Gibco) supplemented with 10% heat-inactivated foetal bovine serum (FBS) (Gibco), 2 mM l-glutamine (Gibco), 1 mM sodium pyruvate (Gibco) and 1% penicillin/streptomycin solution (Gibco). The cells were cultured at 37 °C with 5% CO_2_ in a humidified atmosphere. Cells were plated 24 h before infection in Dulbecco’s modified eagle’s medium (DMEM) (without antibiotics) containing 10% FBS. For infections, bacteria were grown in MB 7H9 broth (Difco) supplemented with 0.05% Tween 80 and 10% ADC. Bacterial clumps were removed by passing through a 26 gauge syringe ten times. Infection was carried out for 4 h, followed by washing and incubation in gentamycin-containing medium for 2 h. The cells were further incubated in DMEM supplemented with 10% FBS for different periods of time.

### CFU determinations

RAW264.7 cells were plated in 48-well plates at a seeding density of 1×10^5^ cells and were grown in DMEM supplemented with 10% FBS overnight. The cells were then infected with *M. tuberculosis* or its genetically manipulated derivatives at an multiplicity of infection (MOI) of 10 as described above. Subsequently, the cells were cultured for different periods of time and then lysed. Serial dilutions of the lysate were plated on MB 7H11 agar plates supplemented with 10% OADC and incubated at 37 °C for 3–4 weeks. The viable bacteria present in the cell lysates were enumerated by counting CFUs.

### RNA isolation from *M. tuberculosis* and quantitative reverse transcription PCR

RNA was isolated from mycobacterial cells (*M. tuberculosis-pMV261*, *M. tuberculosis-pMV261-ydeH* and *M. tuberculosis-pMV261-rv1357c*) using the RNeasy Mini Kit (Qiagen). Briefly, bacteria were lysed by agitation with six pulses of 1 min duration each in a Mini bead beater apparatus (BioSpec Products, USA). The lysates were centrifuged at 10 000 *g* for 15 min at 4 °C, and the supernatant was transferred to a 1.5 ml centrifuge tube. An equal volume of 70% ethanol was added to the lysate, mixed well by pipetting, and RNA was isolated following the manufacturer’s instructions. DNA contamination was removed using Turbo DNase (Ambion, product code: AM1907). cDNA was synthesized from RNA using the RevertAid First Strand cDNA Synthesis Kit (Fermentas). Gene expression analysis was performed using quantitative reverse transcription PCR (qRT-PCR) with KAPA SYBR FAST Master Mix (Kapa Biosystems, USA) and gene-specific primers detailed in Table 3. Melting curve analysis was performed to confirm amplification specificity. Expression of *sigA* was unchanged in all samples. The relative expression of the target gene was normalized to *sigA*. The comparative CT (also known as 2^−∆∆CT^) method was used for analysing gene expression. Fold change was determined relative to the expression level in *Mtb-pMV261*.

**Table 3.** List of primers used for qRT-PCR

Primer name	Primer sequence (5′−3′)
*fbpC*F	AGAGCAACGGCCAGAACTAC
*fbpC*R	CCCGACATCGAAAGACCCAC
*mtrA*F	TCATCGGCGACGGTACTCAG
*mtrA*R	GGGCAGCATCAAATCCAATAACAC
*ripA*F	TGGAAACCGCTCGGGACAAC
*ripA*R	AACGTGTCGAACCGGTGCTG
*esxG*F	CGGCTCAGGCGTTTCAC
*esxG*R	CCGCCGCCACAAACC
*esxH*F	CAGGCCGCGTTGCA
*esxH*R	CTGCCACGCCTGATACGT
*dnaA*F	AGTCCGGTCTCGGCAAGAC
*dnaA*R	TGAAGTCGTTGGTGAATTCCTC
*rv1357c*F	GGTCAAACTCGGGGGAAAGT
*rv1357c*R	GACGGTGATACCGAGCTTGT
*rv1354c*F	GACCTACATACCGCCAGAGC
*rv1354c*R	ACAAAACATTCGTGCGGCAA
*sigA*F	GGTGATTTCGTCTGGGATGAA
*sigA*R	GCTACCTTGCCGATCTGTTTG

F, forward; R, reverse.

### RNA isolation from intracellular bacteria

RAW264.7 cells were seeded at 10^7^ cells per T75 flask in four flasks for each strain of *M. tuberculosis*. The cells were infected at an MOI of 10. RNA was isolated as described earlier [33]. Briefly, the cells were washed by gently shaking the flasks two to three times with sterile PBS. Cells were then lysed by adding 20 ml of guanidine thiocyanate buffer containing *N*-lauryl sarcosine, sodium citrate and *β*-mercaptoethanol. The lysate was centrifuged and the bacterial pellet was resuspended in 1 ml PBS containing 0.1% Tween 80. After centrifugation, the pellet was stored at −80 ˚C until further use. The intracellular bacteria were further lysed by adding lysozyme solution (5 mg ml^−1^ in RNase-free water) and incubating at room temperature for 1 h, with intermittent vortexing. Trizol (pre-warmed to 65 ˚C) was added and thoroughly mixed by inverting the tubes. The tubes were half-filled with 0.1 mm glass beads. Cells were lysed by homogenization using a Mini bead beater apparatus (BioSpec Products, USA). Next, 200 µl of chloroform was added to the lysate, mixed and centrifuged, and the aqueous phase was carefully transferred to a fresh centrifuge tube. An equal volume of 100% ethanol was added to the lysate and mixed well by pipetting. Then, 700 µl of the lysate mixture was transferred to an RNeasy spin column and RNA was isolated using the RNeasy Mini kit (Qiagen) according to the manufacturer’s instructions. The isolated RNA was treated with Turbo DNase (Ambion), and gene expression analysis was performed by qRT-PCR as described above.

### Immunofluorescence microscopy

RAW264.7 cells were grown on coverslips and infected with fluorescein isothiocyanate (FITC) labelled *M. tuberculosis* strains as described by Banerjee *et al*. [8]. The cells were then fixed with 4% paraformaldehyde (v/v) for 10 min, permeabilized with 0.01% Triton X-100 in PBS and treated with 2% bovine serum albumin in PBS. This was followed by an overnight treatment with anti-lysosomal-associated membrane protein 1 (LAMP1) antibody (Abcam) at a dilution of 1:500 at 4 °C. The cells were then treated with Alexa 546-conjugated goat anti-rabbit antibody (Abcam) and the coverslips were mounted with SlowFade (Thermo Scientific). Nuclei were stained with 4′,6-diamidino-2-phenylindole (DAPI). Finally, the slides were imaged using a confocal microscope (TCS SP8, Leica Microsystems, Germany).

### Statistical analysis

Distribution analysis of the data was performed by Shapiro–Wilk test. All data that passed the normality test are presented as means±sd. For the grouped comparisons, Kruskal–Wallis followed by Dunn test or one-way Analysis of Variance (ANOVA) followed by Tukey test was done. All analyses were performed using GraphPad Prism. A *P* value≤0.05 was considered to be significant.

## Results

### C-di-GMP binds to the histidine kinase MtrB of *M. tuberculosis*

**Fig. 1.**
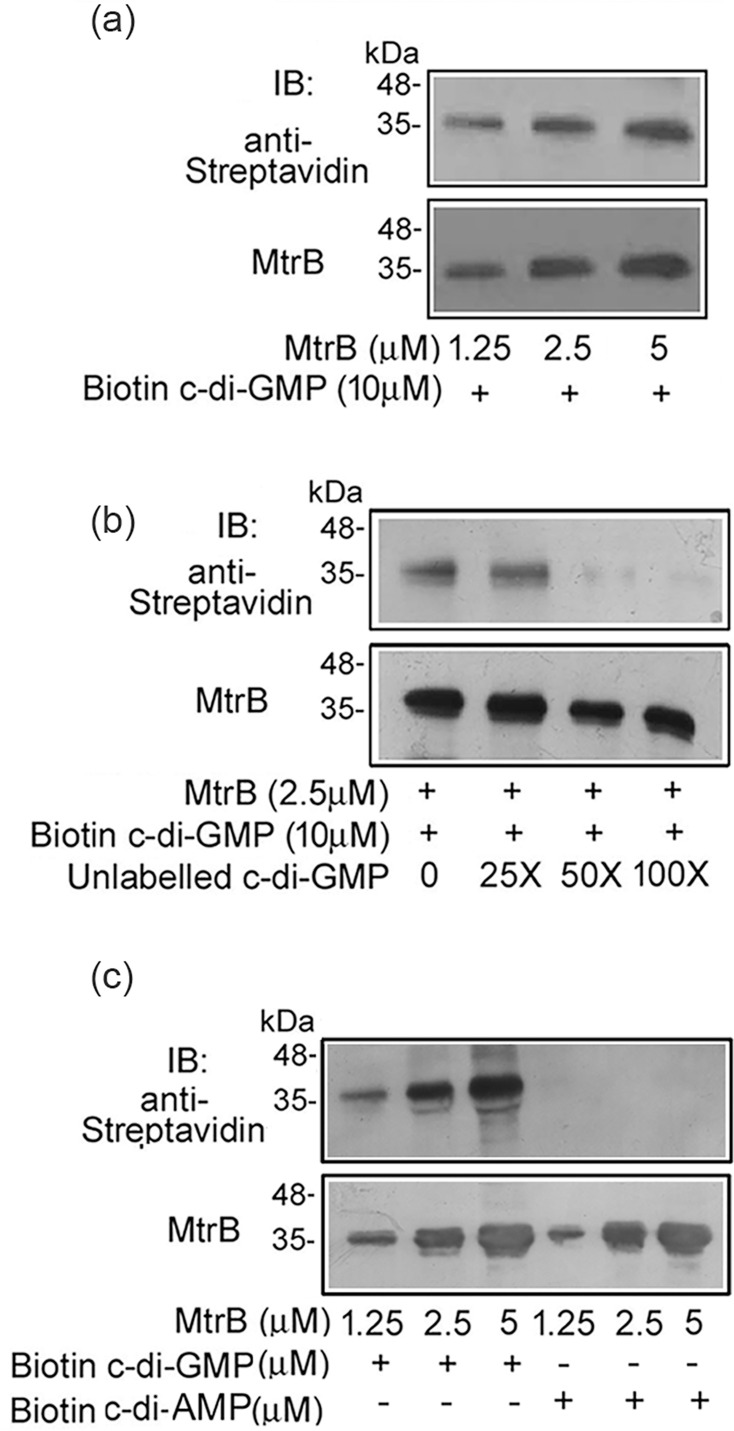
C-di-GMP interacts with MtrB. (**a**) Varying concentrations of MtrB were incubated with biotinylated c-di-GMP and the samples were subjected to cross-linking using ultraviolet light irradiation. Cross-linked samples were immunoblotted with anti-streptavidin antibody and reprobed with MtrB-specific antibody. (**b**) MtrB was incubated with 10 µM biotinylated c-di-GMP in the absence or presence of increasing concentrations of unlabelled c-di-GMP. (**c**) MtrB was incubated with different concentrations of biotinylated c-di-GMP or c-di-AMP. Binding was followed as described under panel A. Blots are representative of three independent biological experiments.

Recent studies have shown that c-di-GMP acts as a signalling molecule that regulates the functions of TCSs from several bacteria, including mycobacteria. Taking together the observations that MtrB regulates biofilm formation in *M. tuberculosis* [8] and that c-di-GMP regulates biofilm formation in a number of bacteria, we asked the question of whether MtrB is subject to regulation by c-di-GMP. We first tested whether c-di-GMP binds to the cytosolic domain of MtrB. In order to do so, the cytosolic domain of MtrB was incubated with biotinylated c-di-GMP, followed by UV cross-linking of the sample, SDS/PAGE and electrotransfer of the proteins. Biotinylated conjugates were detected by incubation with HRP-conjugated streptavidin antibody, followed by chemiluminescence-based detection. It was observed that the cytosolic domain of MtrB interacts with c-di-GMP in a concentration-dependent manner (Fig. 1a). As a positive control, c-di-GMP binding was tested with STING, a c-di-GMP interacting protein [34], and found positive (Fig. S1, available in the online Supplementary Material). The binding of biotinylated c-di-GMP with MtrB could be competed by unlabelled c-di-GMP (Fig. 1b), suggesting the specificity of the interaction. On the other hand, biotinylated c-di-AMP failed to bind MtrB (Fig. 1c), further confirming the specificity of the interaction.

### C-di-GMP regulates MtrB autophosphorylation

**Fig. 2.**
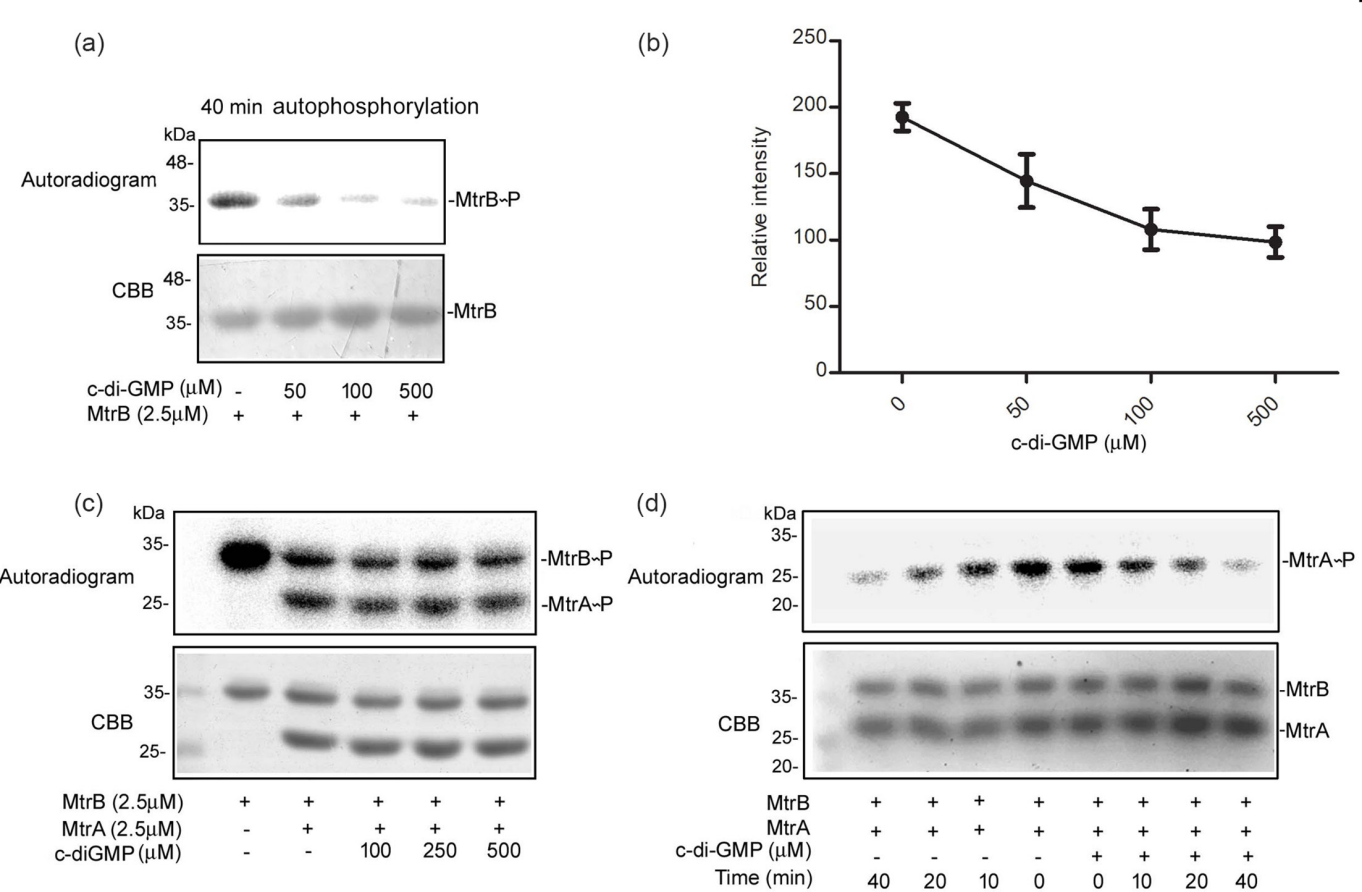
Effect of c-di-GMP on MtrB autophosphorylation, transphosphorylation and phosphatase activities. (**a**) An *in vitro* autophosphorylation reaction was carried out with MtrB in the absence or presence of the indicated concentrations of c-di-GMP for 40 min in autophosphorylation buffer containing [*γ*^−32P^] ATP. Phosphorylated MtrB is indicated as MtrB-P. The top panel shows the autoradiogram and the bottom panel the corresponding Coomassie brilliant blue (CBB) stained gel. (**b**) Densitometric analysis of the data shown in panel A. (**c**) Transphosphorylation of MtrA by radiolabelled MtrB-P in the absence or presence of different concentrations of c-di-GMP. (**d**) For phosphatase activity assays, phosphorylated ([*γ*^−32P^] labelled) MtrA was incubated with non-phosphorylated MtrB for different periods of time in the absence or presence of c-di-GMP (200 µM) and the disappearance of phosphorylated MtrA was monitored by SDS/PAGE and autoradiography. The autoradiograms shown are representative of three independent biological experiments.

Phosphorylated MtrB binds with higher affinity to cognate and non-cognate RRs than its unphosphorylated counterpart [15]. The first step for TCS signalling is autophosphorylation of the SK. We therefore reasoned that regulation of autophosphorylation of MtrB by c-di-GMP could be one of the mechanisms of regulating downstream effects of MtrB. We tested whether c-di-GMP regulates MtrB autophosphorylation. Autophosphorylation of MtrB was performed either in the absence or in the presence of varying concentrations of c-di-GMP. Autophosphorylation of MtrB was inhibited by c-di-GMP in a concentration-dependent manner (Fig. 2a, b). This result suggested that c-di-GMP inhibits the first step in MtrAB signalling. After autophosphorylation, phosphorylated MtrB transfers phosphate to its cognate RR MtrA *in vitro* [30]. We therefore tested the effect of c-di-GMP on the transphosphorylating activity of MtrB using MtrA as substrate. MtrB was phosphorylated with radiolabelled ATP in the absence of c-di-GMP. Phosphorylated MtrB was then incubated with MtrA either in the absence or in the presence of c-di-GMP. Phosphotransfer to MtrA was not altered in the presence of c-di-GMP (Fig. 2c). MtrB is a bifunctional SK [35]. It exhibits phosphatase activity. This has been demonstrated using phosphorylated MtrA as substrate *in vitro*. The effect of c-di-GMP on the ability of MtrB (non-phosphorylated) to dephosphorylate phosphorylated MtrA was therefore tested. C-di-GMP did not alter the phosphatase assay of MtrB (Fig. 2d). In summary, c-di-GMP inhibits the autophosphorylation of MtrB, which is the first step of TCS signalling, but does not alter its ability to transphosphorylate MtrA or to dephosphorylate phosphorylated MtrA.

### C-di-GMP regulates the expression of genes of the MtrA regulon

**Fig. 3.**
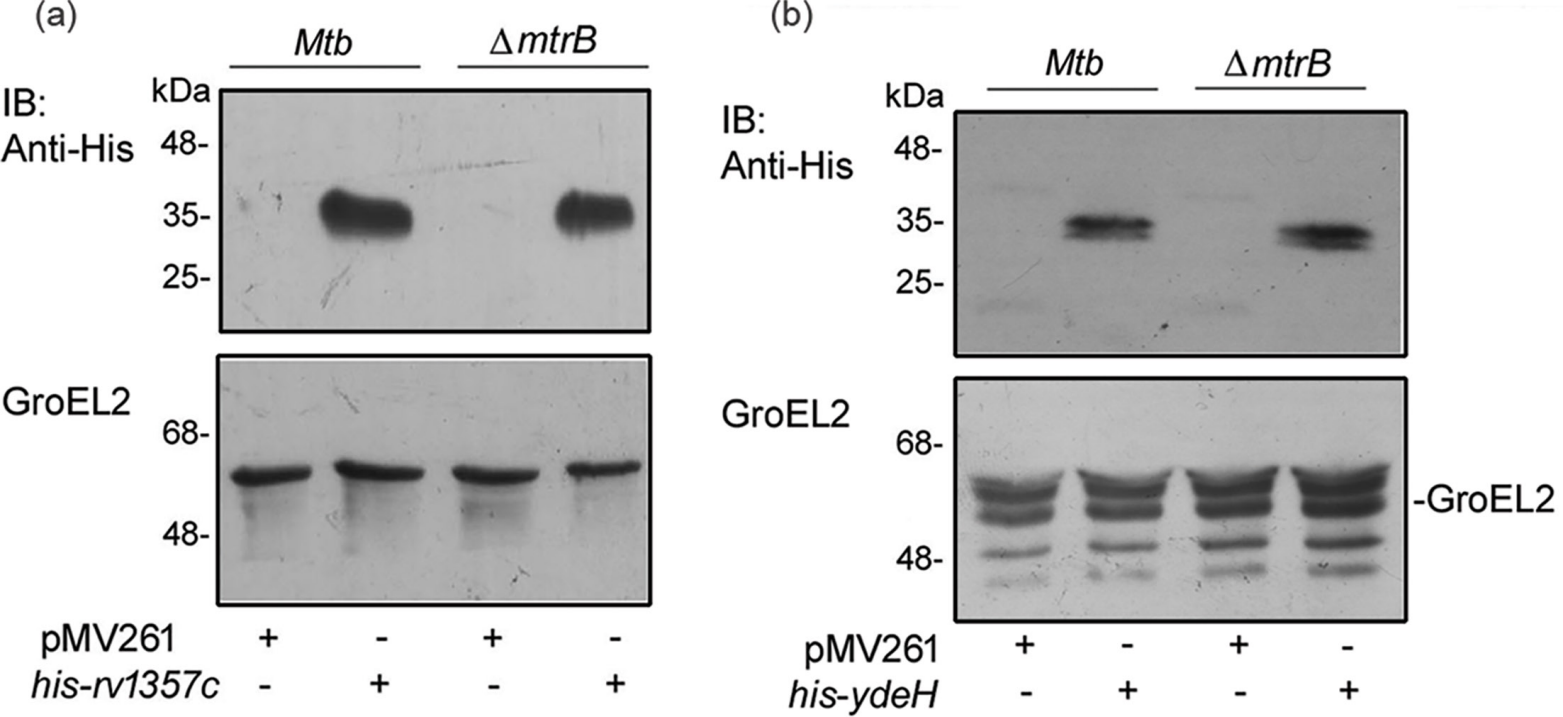
Expression of His-tagged c-di-GMP phosphodiesterase (*rv1357c*) or DGC (*ydeH*) in *M. tuberculosis*. (**a, b**) Overexpression of *his-rv1357c* and *his-ydeH* in *M. tuberculosis* (a) and *∆mtrB* (**b**) was validated by Western blotting of lysates of the respective strains using anti-His antibody. GroEL2 was used to confirm equal loading of lysates. The blots are representative of three independent experiments.

**Fig. 4.**
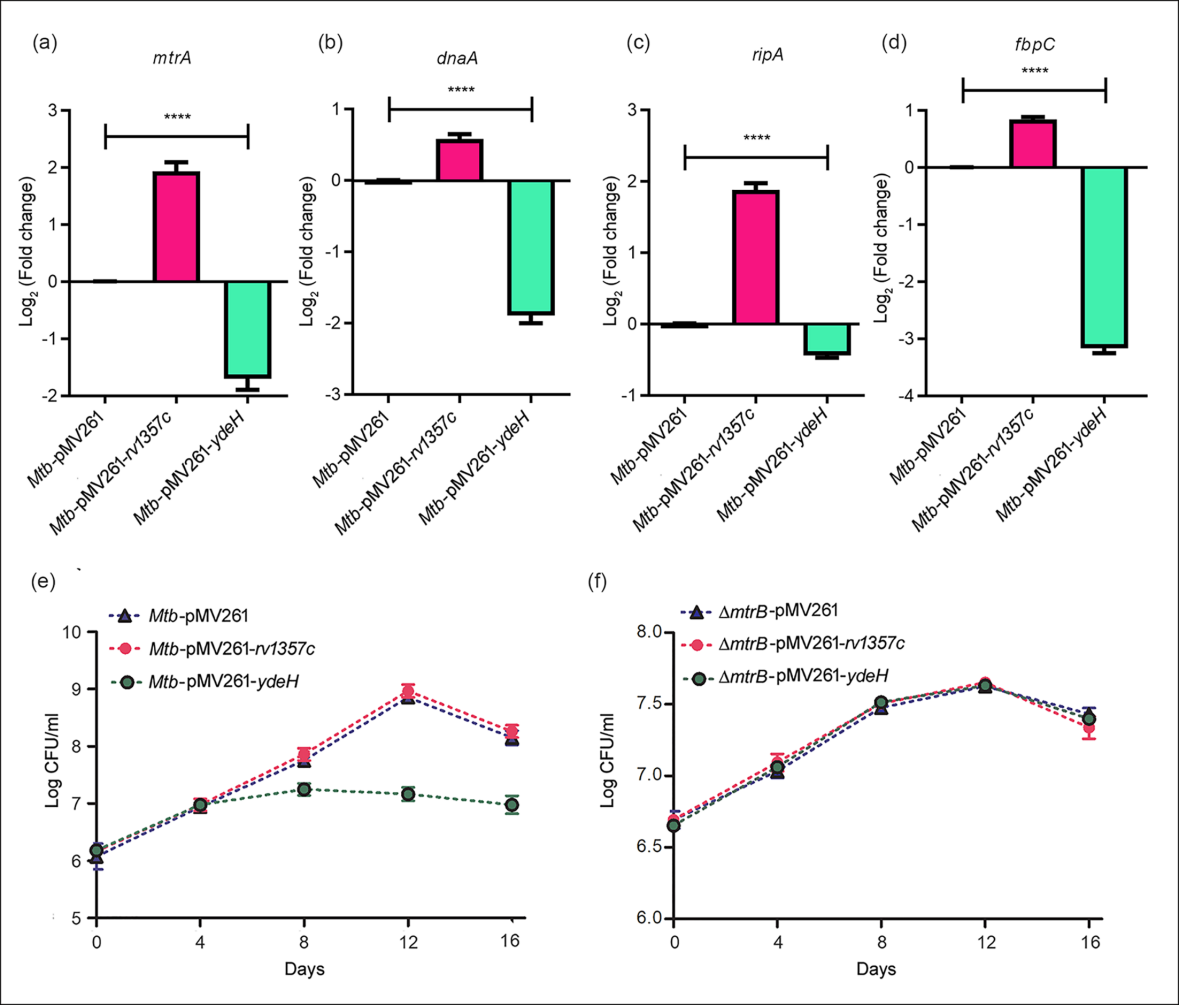
C-di-GMP synthase and phosphodiesterase regulate expression of genes of the MtrA regulon and bacterial survival. (**a–d**) The expression of selected genes of the MtrA regulon along with MtrA was determined by qRT-PCR in *Mtb*-pMV261 and the strains overexpressing the phosphodiesterase (*rv1357c*) [*Mtb*-pMV261-*rv1357c*] or DGC (*ydeH*) [*Mtb*-pMV261-*ydeH*]. The relative expression of target genes was normalized to *sigA* and compared with the expression in *Mtb*-pMV261. The comparisons were made with Kruskal–Wallis followed by Dunn’s test; *n*=6; ****P≤0.0001. (**e, f**) Bacterial strains were grown and CFUs were enumerated at different time points for *Mtb*-pMV261, *Mtb*-pMV261-*rv1357c*, *Mtb*-pMV261-*ydeH* (e) and for *ΔmtrB*-pMV261, *ΔmtrB*-pMV261-*rv1357c*, *ΔmtrB*-pMV261-*ydeH* (**f**). Results are representative of three independent experiments.

C-di-GMP phosphodiesterase deletion in *M. bovis* BCG alters expression of genes of the MtrA regulons [26]. In order to explore the role of c-di-GMP in regulating genes linked to the MtrA regulon of *M. tuberculosis in vivo* through its likely role in MtrB autophosphorylation, the strategy of manipulating c-di-GMP levels in *M. tuberculosis* through overexpression of the c-di-GMP synthesizing and degrading enzymes was employed. C-di-GMP is produced from two GTP molecules by diguanylate cyclase (DGC) containing a GGDEF (or GGEEF) signature motif domain and degraded by phosphodiesterase containing an EAL domain or GYP domain [36]. C-di-GMP levels in *M. tuberculosis* are maintained by two different enzymes, namely DGC (Rv1354c) and phosphodiesterase (Rv1357c), which synthesize and degrade c-di-GMP, respectively [37]. However, the DGC Rv1354c has both synthesizing and degrading activity *in vivo* [37]. Therefore, in order to manipulate the levels of c-di-GMP in *M. tuberculosis*, the *E. coli-*derived DGC *ydeH* [38] and the *M. tuberculosis* phosphodiesterase *rv1357c* were overexpressed in the different * M. tuberculosis* strains as His-tagged proteins generating the *Mtb*-pMV261-*ydeH* and *Mtb*-pMV261-*rv1357c*, respectively. The overexpression of each enzyme was confirmed by Western blotting using anti-His antibody (Fig. 3a, b). It was confirmed that overexpression of *ydeH* (a c-di-GMP synthesizing gene of *E. coli*) did not alter levels of *rv1357c* (a c-di-GMP phosphodiesterase) (Fig. S2). Similarly, overexpression of *rv1357c* did not alter the level of the *M. tuberculosis* c-di-GMP synthesizing gene *rv1354c* (Fig. S2). We next tested the levels of c-di-GMP in the above strains. Cell lysates were subjected to HPLC to analyse the levels of c-di-GMP. The expression of c-di-GMP was higher in *Mtb*-pMV261-*ydeH* and lower in *Mtb*-pMV261-*rv1357c* compared to the wild-type strain (Fig. S3). C-di-GMP peaks in the lysates were identified based on their retention time compared to the retention time of standard c-di-GMP. The concentrations for c-di-GMP were 11, 5 and 63 µM in *Mtb-*pMV261, *Mtb*-pMV261-*rv1357c* and *Mtb*-pMV261-*ydeH*, respectively. Having confirmed that c-di-GMP levels had successfully been manipulated after overexpression of c-di-GMP synthesizing and degrading enzymes, these strains were further tested for the relative expression of genes of the MtrA regulon [12,39] since phosphorylation of MtrA through MtrB enhances the DNA binding ability of MtrA [15,40]. Previous studies have shown that the promoters of the cell wall hydrolase *ripA* (*rv1477*), the cell wall mycolyl transferase *fbpB* (*rv1886c*) and the replication initiator *dnaA* (*rv0001*) are MtrA targets [9,11]. MtrA binds to its own promoter and is expected to be autoregulated. Furthermore, phosphorylated MtrA binds with greater efficiency than its non-phosphorylated counterpart [10]. An increase in the level of phosphorylation of MtrB reportedly correlates with the levels of phosphorylated MtrA [30]. Therefore, it was logical to assume that modulation of MtrB phosphorylation by c-di-GMP could regulate MtrA phosphorylation and subsequent transcription of MtrA as well as its regulon. qRT-PCR showed that *mtrA* and several MtrA-regulated genes, namely *ripA*, *fbpC* and *dnaA*, as well as *mtrA* itself, are differentially regulated upon overexpression of either c-di-GMP synthase or phosphodiesterase in * M. tuberculosis*. The aforesaid genes were upregulated upon overexpression of the c-di-GMP phosphodiesterase *rv1357c* (Fig. 4a–d) and downregulated upon overexpression of the c-di-GMP synthase *ydeH* (Fig. 4a–d), compared to the levels of expression of each gene in *M. tuberculosis*-pMV261. This was in support of previous observations in *M. bovis* BCG showing that deletion of c-di-GMP phosphodiesterase is associated with transcriptional attenuation of the *mtrA* regulon [26]. Overexpression of c-di-GMP phosphodiesterase causes a reduced level of c-di-GMP compared to the wild-type *M. tuberculosis*. We hypothesize that the reduced level of c-di-GMP is associated with increased levels of phosphorylated MtrB. This in turn likely leads to an increased level of phosphorylated MtrA leading to enhanced activation of MtrA and its regulon. Conversely, increased levels of c-di-GMP lead to attenuation of MtrB autophosphorylation and reduced MtrA activity, culminating in reduced expression of MtrA regulon genes.

An earlier report has demonstrated that MtrA is essential for bacterial growth *in vitro* [41]. Since we observed that manipulation of intracellular c-di-GMP alters the transcription of MtrA, it was logical to assume that it may affect the growth of the bacterium. Therefore, we tested the growth of the respective bacterial strains with altered levels of intracellular c-di-GMP compared to the wild-type strain. It was observed that overexpression of *ydeH* attenuates the growth of the bacterium compared to the parent strain or the strain overexpressing *rv1357c* (Fig. 4e). In order to ensure that the role of c-di-GMP is mediated through regulation of MtrB, the *mtrB*-inactivated strain *ΔmtrB* was employed [8]. Both *ydeH* and *rv1357c* were overexpressed in *ΔmtrB* (Fig. 3a, b). No effect on growth was observed in *ΔmtrB* overexpressing either *rv1357c* or *ydeH* compared to *ΔmtrB* (Fig. 4f). These results suggested that the effects on growth as a consequence of tuning c-di-GMP levels are largely mediated through MtrB. This result was in harmony with our hypothesis that reduced levels of MtrA due to c-di-GMP-dependent negative regulation of phosphorylation of MtrB are linked to reduced bacterial growth *in vitro*. It is plausible that the effect on bacterial growth is mediated through MtrA since MtrA regulates genes linked to cell wall biosynthesis, cell division and replication [11,14].

### C-di-GMP levels regulate *M. tuberculosis* biofilm formation in an MtrB-dependent manner

C-di-GMP regulates biofilm formation in bacteria such as *Glutamicum xylinus*, *Salmonella enterica*, *Vibrio cholerae and P. aeruginosa* [18]. In *P. aeruginosa*, the GacS/GacA TCS, as well as c-di-GMP, regulates biofilm formation [42]. In *M. bovis* BCG, alterations of c-di-GMP regulate biofilm formation [26,43].

**Fig. 5.**
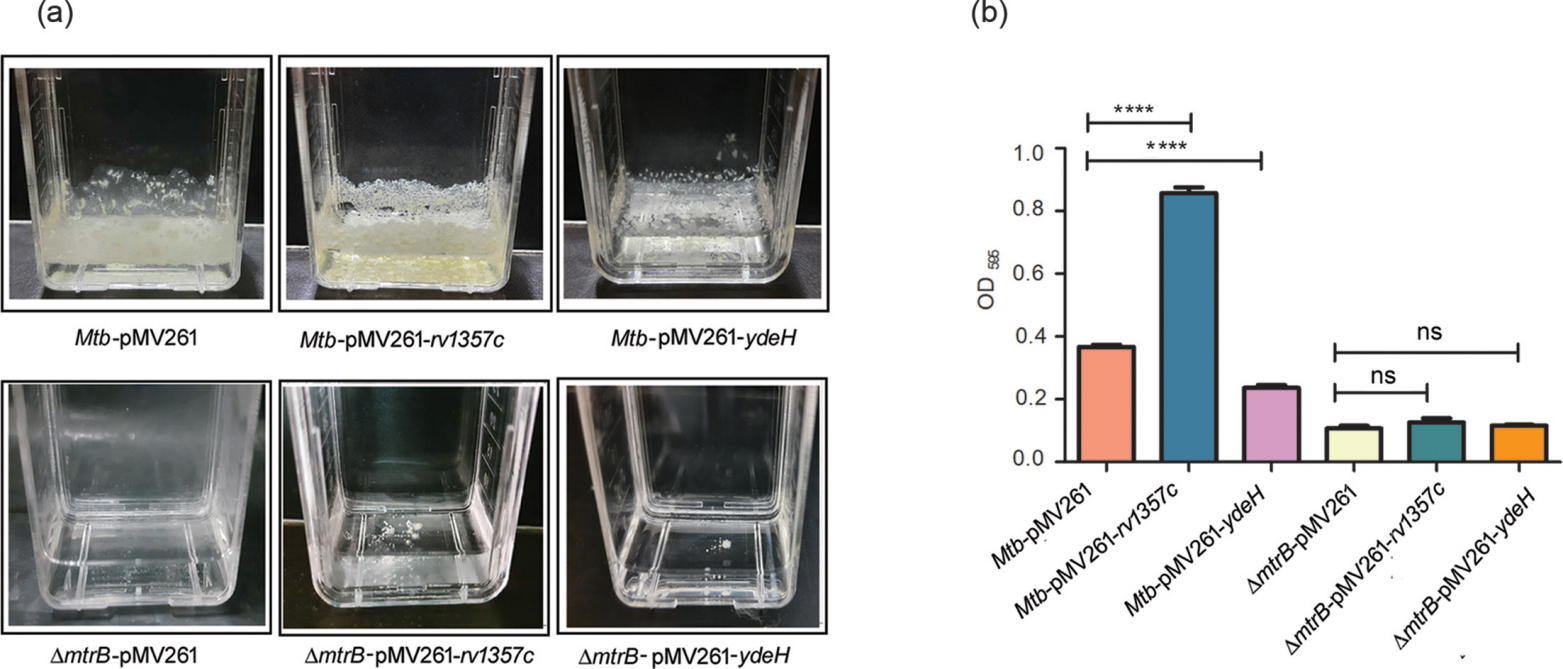
Biofilm formation in *M. tuberculosis* is regulated by c-di-GMP. (**a**) Biofilm formation of *Mtb*-pMV261, *Mtb*-pMV261-*rv1357c*, *Mtb*-pMV261-*ydeH*, *ΔmtrB*-pMV261, *ΔmtrB*-pMV261-*rv1357c*, *ΔmtrB*-pMV261-*ydeH* after 6 weeks of incubation. Panel (**b**) is a quantitative representation of the CV taken up by biofilms of the different strains shown in panel A. Data were analysed using one-way ANOVA followed by Tukey’s test; ns, non-significant; ****P<0.0001. Images in panel A and the data in panel B are representative of at least three independent experiments performed with three technical replicates each.

Our previous study has shown that MtrB is essential for biofilm formation [8]. We tested whether alterations in c-di-GMP levels influence biofilm formation in an MtrB-dependent manner. We observed that overexpression of *rv1357c*, which is associated with decreased levels of c-di-GMP, led to increased biofilm formation, whereas overexpression of *ydeH*, which is associated with increased levels of c-di-GMP, led to reduced biofilm formation compared to the parent strain (Fig. 5a, b). Bacterial viability in biofilms was also determined by enumerating CFUs. It was observed that overexpression of *ydeH* led to reduced CFUs, whereas overexpression of *rv1357c* augmented CFUs (Fig. S4). However, expression of neither *rv1357c* nor *ydeH* could rescue the biofilm defective phenotype of *ΔmtrB* (Fig. 5a, b), suggesting that c-di-GMP exerts its role on biofilm formation through MtrB.

### C-di-GMP tunes intracellular survival of *M. tuberculosis* and expression of genes of the *mtrA* regulon

**Fig. 6.**
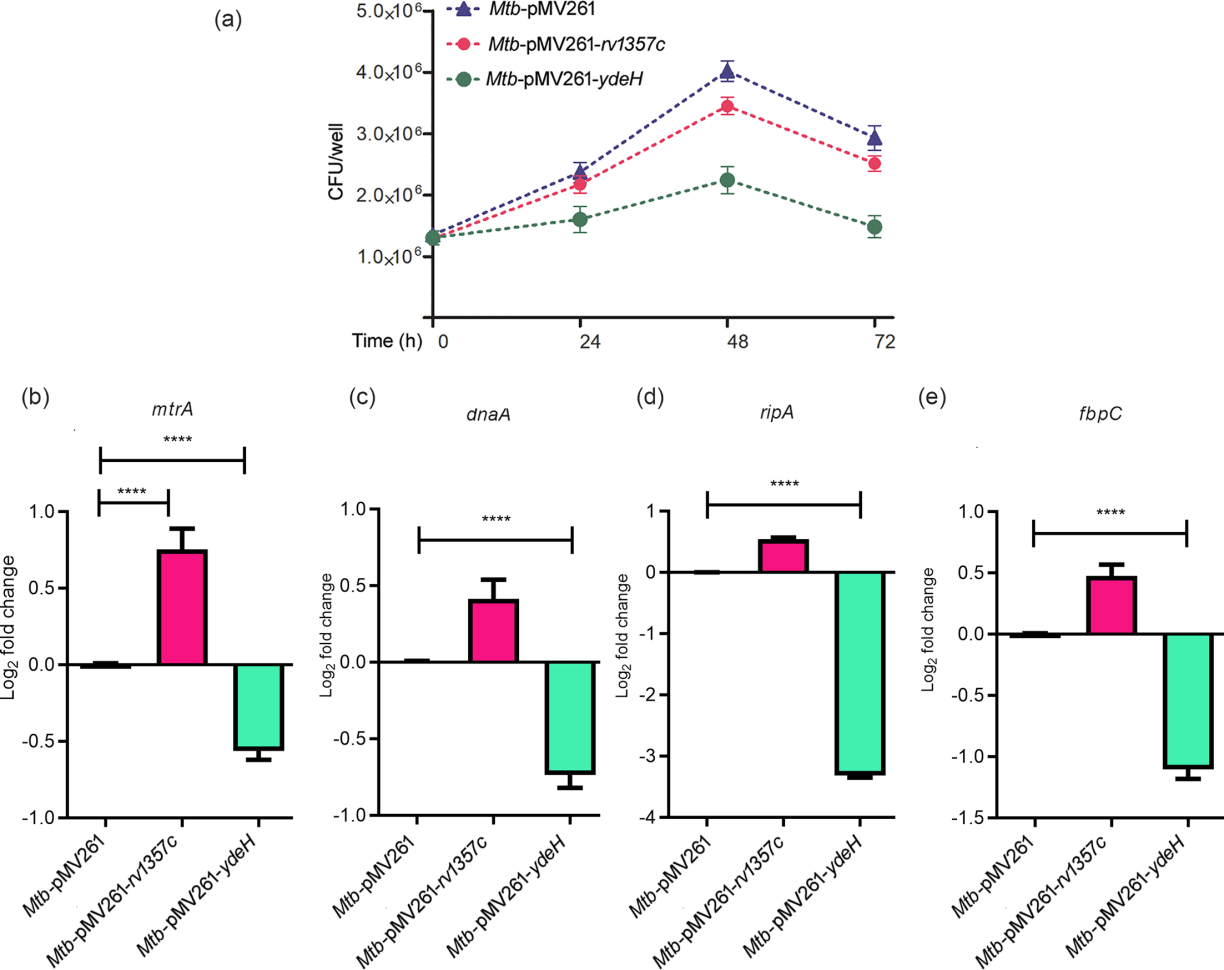
C-di-GMP regulates the survival of *M. tuberculosis* and expression of genes of the MtrA regulon, in macrophages. (**a**) RAW264.7 cells were infected with *M. tuberculosis*-pMV261 or its derivatives. Cells were lysed at different times of infection and bacterial survival was determined by enumerating CFUs. Results are representative of three independent experiments. (**b–e**) Macrophages were infected with *M. tuberculosis*-pMV261 and mycobacterial strains overexpressing the phosphodiesterase (*rv1357c*) [*Mtb*-pMV261-*rv1357c*] or DGC (*ydeH*) *[Mtb*-pMV261-*ydeH*]. RNA was isolated from intracellular bacteria and relative expression of selected genes was determined by qRT-PCR. The relative expression of target genes was normalized to *sigA* and compared with the expression in *Mtb*-pMV261. Data were analysed using one-way ANOVA followed by Tukey’s test (**b**) or Kruskal–Wallis followed by Dunn’s test (**c–e**); *n*=6; ****P<0.0001.

In order to test whether modulation of c-di-GMP levels impacts the survival of *M. tuberculosis* in macrophages, RAW264.7 cells were infected with *M. tuberculosis-pMV261*, *M. tuberculosis-pMV261-rv1357c* or *M. tuberculosis-pMV261-ydeH* and survival of these strains was assessed by determining CFUs. When compared to the parent strain, intracellular growth was compromised upon overexpression of *ydeH* (Fig. 6a), which is associated with enhanced c-di-GMP levels. This particular finding was in harmony with previous observations where deletion of c-di-GMP phosphodiesterase or higher accumulation of cellular c-di-GMP in *M. bovis* BCG showed attenuated survival of the bacteria inside macrophages post-internalization [43,44]. On the other hand, overexpression of *rv1357c*, which is associated with reduced levels of c-di-GMP, did not affect growth compared to the wild-type strain (Fig. 6a). We further tested the expression of *mtrA* and genes of the *mtrA* regulon in *M. tuberculosis* residing in macrophages. Similar to the observations made during growth *in vitro*, elevated levels of c-di-GMP in relation to the parent strain were associated with lower levels of expression of *mtrA*, *dnaA*, *ripA* and *fbpC* (Fig. 6b–e), whereas attenuated levels of c-di-GMP were associated with increased levels of expression of the aforesaid genes. These results suggested that c-di-GMP regulates the *mtrA* regulon both in *M. tuberculosis* grown *in vitro* as well as in bacteria residing in macrophages.

### C-di-GMP regulates the trafficking of *M. tuberculosis* to lysosomes

**Fig. 7.**
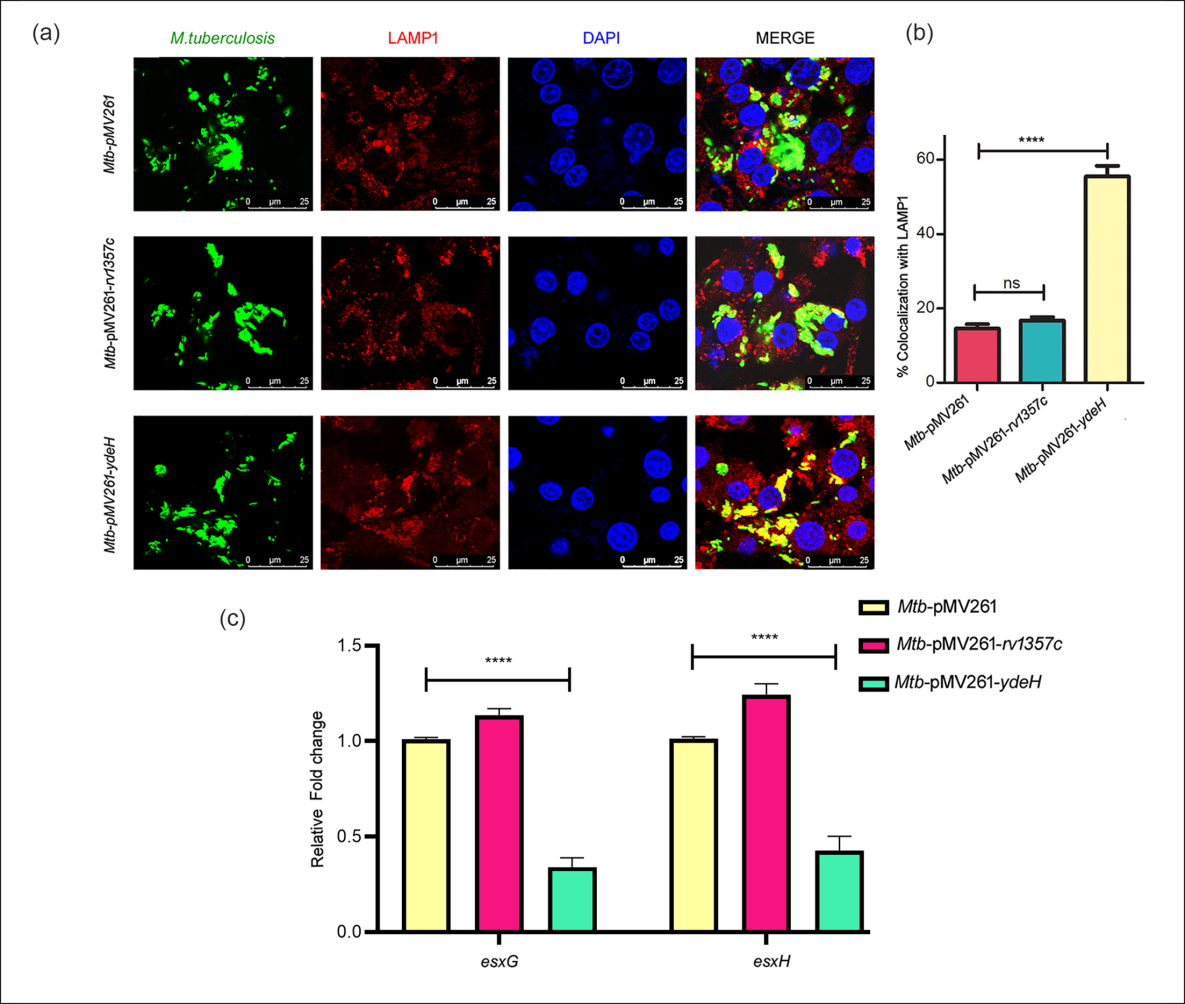
C-di-GMP regulates the trafficking of *M. tuberculosis* to lysosomes and transcription of *esxG* and *esxH*. (**a, b**) RAW264.7 cells were infected with FITC-labelled (green) *Mtb*-pMV261, *Mtb*-pMV261-*rv1357c* and *Mtb*-pMV261-*ydeH*, fixed and immunolabelled with anti-LAMP1 antibody followed by Alexa 546-conjugated secondary antibody (red). The cells were stained with DAPI to visualize the nuclei. The colocalization of green and red fluorescence indicates bacterial trafficking to the lysosomal compartment (**a**). (**b**) Percent colocalization of each of the three strains was calculated by counting at least 100 bacteria from three different fields. Data represent means with sd. *n*=9 including technical replicates. Data were analysed using one-way ANOVA followed by Tukey’s test. ns, non-significant; ****P<0.0001. Scale bar denotes 20 µM. Three independent experiments were carried out. (**c**) Relative expression of genes associated with lysosomal trafficking, *esxG* and *esxH*, was quantitated by qRT-PCR in *Mtb*-pMV261 and the mycobacterial strain overexpressing the phosphodiesterase (*rv1357c*) [*Mtb*-pMV261-*rv1357c*] and the DGC [*Mtb*-pMV261-*ydeH*] after infection. The relative expression of target genes was normalized to *sigA* and compared with the expression in *Mtb*-pMV261. Data were analysed using Kruskal–Wallis followed by Dunn’s test; *n*=6; ****P<0.0001.

The pathogenicity of *M. tuberculosis* is directly linked to the ability of the bacterium to survive in macrophages. The bacterium uses a number of mechanisms to avoid phagolysosomal fusion and thereby evade clearance from within its host. Our previous studies [8] have shown that trafficking of *M. tuberculosis* to lysosomes was enhanced in the absence of MtrB. Considering that overexpression of *ydeH* compromised the growth of *M. tuberculosis* in macrophages (Fig. 6a), we tested the trafficking of this strain to lysosomes in comparison with *M. tuberculosis-pMV261*. Overexpression of *ydeH* enhanced trafficking to lysosomes (Fig. 7a, b). On the other hand, overexpression of *rv1357c* did not alter trafficking to lysosomes in comparison with the parent strain (Fig. 7a, b). Considering that c-di-GMP attenuates MtrB autokinase activity, these results were in harmony with the observations of Banerjee *et al*. [8] that inactivation of MtrB leads to enhanced trafficking to lysosomes. Trafficking to the lysosome is facilitated by the ESCRT machinery. EsxG and EsxH, components of the ESX-3 secretion system of *M. tuberculosis*, disrupt ESCRT function [45], and MtrB regulates EsxG and EsxH [8]. As expected, *esxG* and *esxH* were downregulated when *ydeH* was overexpressed (Fig. 7c). This was in harmony with our previous observation that MtrB is required for expression of *esxG* and *esxH* [8]. The downregulation of *esxG* and *esxH* upon overexpression of *ydeH* is possibly linked to its diminished ability to subvert the host phagolysosomal fusion pathway and to survive in macrophages.

## Discussion

Among the challenges of managing TB through the development of new drugs is the incomplete understanding of the sensing mechanisms utilized by the bacterium to respond to changes in the extracellular milieu. The interplay between various signalling systems of *M. tuberculosis* is poorly understood. TCSs represent one of the major signalling systems that enable *M. tuberculosis* to sense and respond to conditions prevailing within the host environment. Among the TCSs, our previous studies have shown that the SK MtrB, a component of the MtrAB TCS, is a central player in the response of the bacterium to hypoxia and to acid stress, in the formation of biofilms and in the subversion of trafficking of the bacterium to lysosomes [8]. Furthermore, it is required for the establishment of infection *in vivo* and efficient granuloma formation within the host lungs [8]. A CRISPR interference chemical-genetics platform to regulate the expression of *M. tuberculosis* genes and analyse bacterial fitness in the presence of different drugs has shown that *mtrA* and *mtrB* are two of the most sensitizing hits for the drugs rifampicin, vancomycin and bedaquiline [46]. In this backdrop, it is of obvious importance to understand the regulation of MtrAB signalling.

Cyclic di-nucleotide signalling regulates stress response pathways [47]. Previous reports have shown that cyclic nucleotides regulate the functions of TCSs, acting both upon RRs such as DosR [48] and SKs such as PdtaS [27]. In *M. smegmatis*, c-di-GMP functions as a coactivator of DosR [48]. C-di-GMP binds DosR and regulates the expression of stress-responsive genes. Furthermore, the binding of c-di-GMP to the SK PdtaS of *M. tuberculosis* perturbs the signalling of the PdtaS-PdtaR TCS [27]. This regulates the adaptation of mycobacteria to nutrient deprivation. Binding of c-di-GMP to the pseudoreceiver domain Rec1 of the SK ShkA of *C. crescentus* leads to liberation of the Rec2 domain, facilitating catalysis [49]. It is also important to mention that c-di-GMP levels are regulated by the activities of both c-di-GMP synthesizing as well as degrading enzymes [36].

Based on these reports, we explored whether c-di-GMP regulates the functions of MtrB. In this communication, we present evidence that c-di-GMP, but not c-di-AMP, is a ligand of MtrB. SKs encompass a dimerization and histidine phosphorylation domain and a catalytic ATP binding domain [50]. We demonstrate that c-di-GMP inhibits the autophosphorylation of MtrB, which is the first step required for initiation of MtrAB signalling and is required for the downstream effects of MtrB on MtrA. In the case of MtrB, autophosphorylation at histidine 305 occurs *in cis* [51]. ATP bound to the catalytic domain phosphorylates histidine 305 of the same molecule. In the absence of a crystal structure of MtrB, we failed to successfully perform molecular docking analyses and identification of the c-di-GMP binding site of MtrB. Our present studies have not explored whether c-di-GMP precludes the binding of ATP to the catalytic domain or its subsequent transfer to histidine 305. This awaits further investigation.

In order to elucidate the likely role of c-di-GMP in regulating MtrB functions, the c-di-GMP synthesizing and degrading enzymes, *ydeH* and *rv1357c*, respectively, were overexpressed in *M. tuberculosis*, and the differential regulation of c-di-GMP in the resultant genetically manipulated strains was confirmed. It was confirmed by qRT-PCR that the MtrA-regulated genes *ripA*, *fbpC* and *dnaA* were upregulated upon overexpression of *rv1357c* (Fig. 4) and downregulated upon overexpression of *ydeH* (Fig. 4), compared to the levels of expression in the parent strain, suggesting that c-di-GMP regulated MtrB activity acts downstream on MtrA regulon genes linked to cell wall synthesis, cell division and replication, thereby regulating bacterial growth.

Organized biofilm-like structures promote bacterial persistence in the face of a regimen of drugs [52], and biofilms are associated with a drug-tolerant phenotype in *M. tuberculosis* [53]. Chakraborty *et al*. [54] have demonstrated that *M. tuberculosis* strains defective in biofilm formation are attenuated for survival in mice, suggesting a protective role of biofilms in immune evasion. Biofilm-like cords form early during *M. tuberculosis* infection of the lungs [55]. A previous study from our laboratory has demonstrated the inability of *ΔmtrB* to form biofilms [8]. Considering this, we tested biofilm formation in the c-di-GMP manipulated strains. It was observed that overexpression of *rv1357c*, which is associated with decreased levels of c-di-GMP, led to increased biofilm formation, whereas overexpression of *ydeH*, which is associated with increased levels of c-di-GMP, led to reduced biofilm formation compared to the parent strain (Fig. 5). However, neither of the two strains could rescue the biofilm defective phenotype of *ΔmtrB*, suggesting that c-di-GMP exerts its role on biofilm formation through MtrB. It may be mentioned that, unlike our present observation, c-di-GMP has been documented to be required for biofilm formation in *M. bovis* BCG [44]. Biofilm formation is a complex process dependent on a number of genes that control the cell surface architecture of mycobacteria. As of date, comprehensive knowledge of these genes, and whether or not they are regulated by c-di-GMP, is lacking in * M. tuberculosis*. In view of this, the reasons for the difference between the effect of c-di-GMP on biofilm formation in *M. bovis* BCG and *M. tuberculosis* are not readily explainable without further investigations.

Bacterial survival in macrophages was attenuated upon overexpression of *ydeH* (Fig. 6), as was the expression of MtrA regulon genes (Fig. 6). It is well established that *M. tuberculosis* survives successfully in macrophages by preventing autophagy and fusion with lysosomes. This is facilitated in part by disruption of the ESCRT machinery by EsxG and EsxH [45]. Interestingly, we observed that manipulation of c-di-GMP levels was associated with alteration of trafficking of *M. tuberculosis* to lysosomes. Overexpression of *ydeH* enhanced colocalization of *M. tuberculosis* with LAMP1, a lysosomal marker, compared to the parent strain (Fig. 7), whereas overexpression of *rv1357c* did not impact lysosomal trafficking. Based on our earlier observations that downregulation of *esxG* and *esxH* in *ΔmtrB* is linked to its diminished ability to subvert the host phagolysosomal fusion pathway, we tested the expression of *esxG* and *esxH* in the *ydeH*-overexpressing strain. It was confirmed that *esxG* and *esxH* expression was attenuated in the *ydeH*-overexpressing strain.

**Fig. 8.**
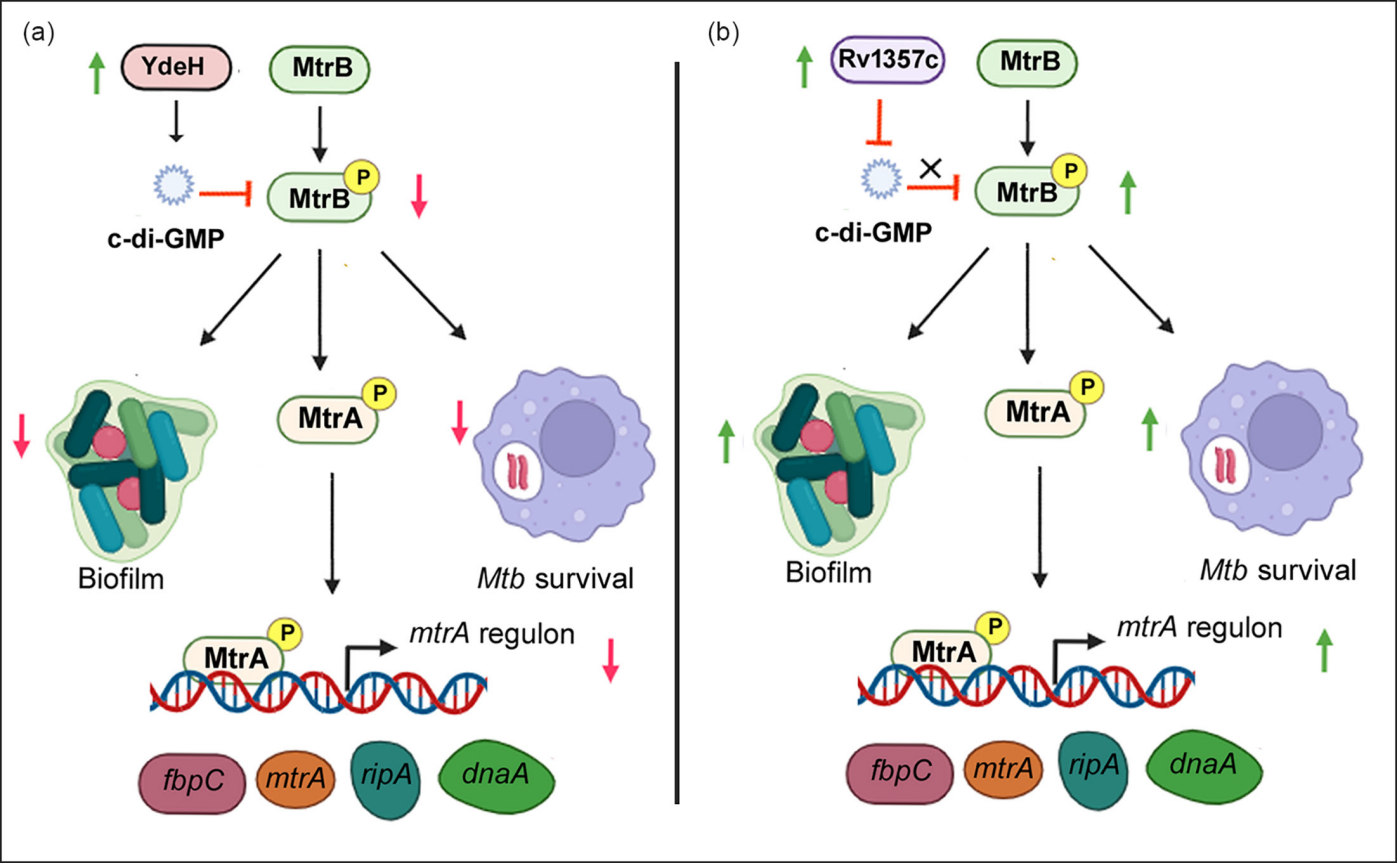
Schematic representation of the crosstalk between c-di-GMP and MtrB and its effects on the *mtrA* regulon, biofilm formation and intracellular survival of *M. tuberculosis*. The overexpression of the DGC *ydeH* (a), leads to enhanced cellular c-di-GMP levels, which attenuates MtrB autophosphorylation. Its downstream effects are reduced expression of MtrA and genes of the MtrA regulon, reduced biofilm formation and decreased intracellular survival of the bacterium. Conversely, overexpression of the c-di-GMP phosphodiesterase *rv1357c* (b), leads to decreased levels of cellular c-di-GMP and enhanced MtrB autophosphorylation. This is associated with activation of the MtrA regulon and enhanced biofilm formation.

In conclusion, our study highlights the interplay between signalling by the cyclic di-nucleotide c-di-GMP and the SK MtrB and its likely role in *M. tuberculosis* infection (Fig. 8). Till now, there is no report elucidating the identity of the signal that regulates MtrB activity and subsequently the MtrA regulon. Our results extend the knowledge of the physiological role of c-di-GMP in * M. tuberculosis*. The c-di-GMP–MtrB axis regulates the autophosphorylation activity of MtrB to impact (a) growth and expression of genes of the MtrA regulon, (b) biofilm formation and (c) trafficking of the bacterium to lysosomes as well as bacterial survival in macrophages. The knowledge gained on c-di-GMP-dependent regulation of MtrB, a mycobacterial SK already shown to be a central regulator of mycobacterial physiology and drug tolerance, unravels a promising pathway for understanding the signalling mechanisms in this pathogen. Alteration of c-di-GMP content in *M. bovis* BCG has been explored to develop new vaccine candidate, with deletion of the c-di-GMP phosphodiesterase leading to a reduction in TB pathology [56,57]. In harmony with an important role of c-di-GMP–MtrB signalling in *M. tuberculosis* physiology and likely in TB pathology, inactivation of *mtrB* is also associated with attenuated lung pathology in infected mice [8].

The current research broadens our understanding of the role of interplay of c-di-nucleotides with TCSs, important cellular signalling components, in the pathogenesis of TB. It expands our knowledge of the c-di-GMP–protein interaction network in mycobacteria. Exemplary of the role of c-di-GMP in mycobacteria, c-di-GMP regulates HpoR [23] and Lsr2 [24] in *M. smegmatis*; the MtrA, ArgR and DosR regulons in *M. bovis* BCG [26]; and PdtaS [27] and EthR [58] in *M. tuberculosis*. In addition, proteome microarray [59] followed by kinetic parameter analyses showed strong interaction of proteins such as Rv1525 (Wbbl2), a putative rhamnosyl transferase, Rv3765c (ProZ) [a component of an ABC transporter] [60] and the peptidoglycan hydrolase Rv1478 [61], with c-di-GMP. The relevance of these interactions is yet to be elucidated.

In order to understand the fine tuning of signalling pathways dependent on TCSs, it is necessary to understand the regulation of TCSs, specifically the role of post-translational modifications and binding of small molecules on the functions of RRs and SKs. One of the focal points of our laboratory has been understanding the role of MtrAB in mycobacterial physiology. Here, we show for the first time that the SK MtrB is regulated by the cyclic di-nucleotide c-di-GMP. Our study needs to be further extended in future towards deciphering the binding pocket of c-di-GMP and deciphering the relevance of this interaction in *in vivo* infection models. It is also pertinent to point out that there is a need to explore other potential regulators of MtrB function. The current research highlights the impact of c-di-GMP–MtrB interaction on *M. tuberculosis* physiology and underscores the potential of intervention of c-di-nucleotide–SK interaction as a strategy for future drug development.

## Supplementary material

10.1099/mic.0.001532 Uncited Supplementary Material 1.

## Abbreviations

ADC: albumin-dextrose-catalase
ANOVA: Analysis of Variance
BCG: Bacillus Calmette Guerin
BSA: bovine serum albumin
c-di-AMP: cyclic di-adenosine monophosphate
c-di-GMP: cyclic-di-guanosine monophosphate
c-di-nucleotides: cyclic di-nucleotides
CFU: colony-forming unit
CV: crystal violet
DAPI: 4′,6-diamidino-2-phenylindole
DMEM: Dulbecco’s modified eagle’s medium
FBS: foetal bovine serum
FITC: fluorescein isothiocyanate
GTC: guanidine thiocyanate
HPLC: high-performance liquid chromatography
HRP: horseradish peroxidase
IPTG: isopropyl *β*-d-1-thiogalactopyranoside
LAMP1: lysosomal-associated membrane protein 1
LBA: Luria−Bertani agar
LBM: Luria−Bertani Miller
MDR: multidrug-resistant
MOI: multiplicity of infection
MWCO: molecular weight cut-off
OADC: oleic acid albumin dextrose catalase
PBS: phosphate-buffered saline
PVDF: polyvinylidene fluoride
qRT-PCR: quantitative reverse transcription polymerase chain reaction
RRs: response regulators
SDS/PAGE: sodium dodecyl sulphate–polyacrylamide gel electrophoresis
SKs: sensor kinases
TB: tuberculosis
TCSs: two-components systems
