# Supplementary material for: Crosstalk between cyclic-di-guanosine monophosphate and the sensor kinase MtrB regulates MtrA-dependent genes, bacterial growth, biofilm formation and lysosomal trafficking of Mycobacterium tuberculosis
Source: Microbiology (Reading). 2025 Feb 7;171(2):001532. doi: 10.1099/mic.0.001532 (PMC12282275; doi:10.1099/mic.0.001532)
Supplement: Uncited Supplementary Material 1. [file mic-171-01532-s001.pdf]

SUPPLEMENTARY INFORMATION

Title: Crosstalk between cyclic diguanosine monophosphate and the sensor kinase MtrB regulates MtrA-dependent genes, bacterial growth, biofilm formation and lysosomal trafficking of *Mycobacterium tuberculosis*

Authors: Shreya Bagchi[1,*], Arun Kumar Sharma[1,*], Soumya Mal[2], Manikuntala Kundu[1,#] and Joyoti Basu[1,#]

Affiliations: [1]Department of Chemical Sciences, Bose Institute, 93/1 APC Road, Kolkata 700009, India

[2]Department of Biological Sciences, Bose Institute, Unified Academic Campus

EN 80, Sector V, Bidhan Nagar, Kolkata 700091, India

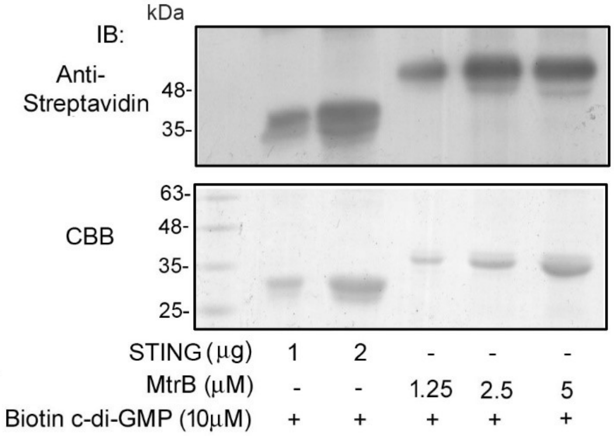

Fig. S1. Binding of the cytosolic domain of MtrB and STING with c-di-GMP. Varying concentrations of MtrB or STING (positive control) were incubated with biotinylated c-di-GMP and the samples were subjected to crosslinking using ultraviolet irradiation. Cross-linked samples were immunoblotted with anti-streptavidin antibody. The bottom panel shows the Coomassie Blue stained gel for MtrB and STING used in the upper panel.

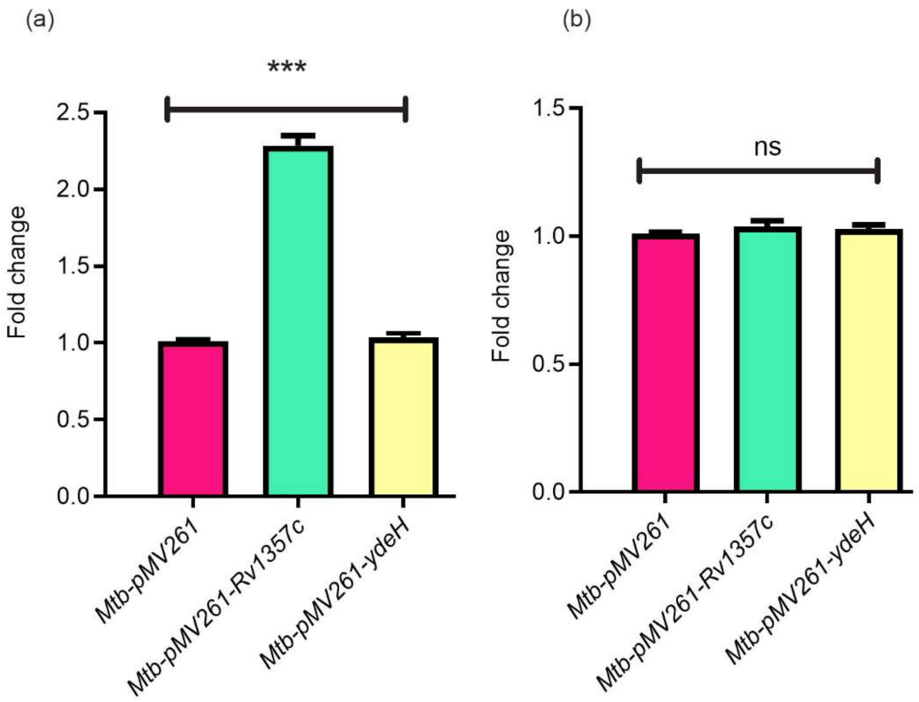

Fig. S2. Expression of *rv1357c* and *rv1354c* in the genetically manipulated strains of *M. tuberculosis. M. tuberculosis.* The expression of *rv1357c* (a) and *rv1354c* (b) was determined by qRT-PCR in *Mtb-pMV261* and the strains overexpressing the phosphodiesterase (*rv1357c*) [*Mtb-pMV261-rv1357c*] or diguanylate cyclase (*ydeH*) [*Mtb-pMV261-ydeH*]. The relative expression of target genes was normalized to *sigA* and compared with the expression in *Mtb-pMV261.* Comparisons were made using Kruskal Wallis followed by Dunn's test. n=6; ns-non significant; ***, P<0.0001.

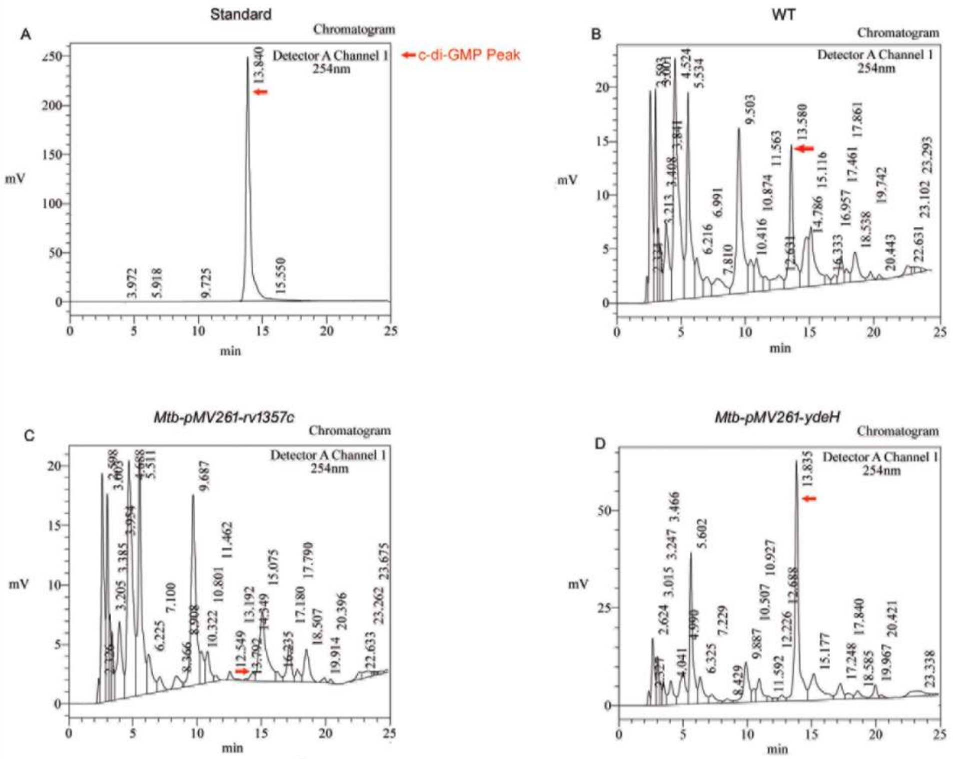

E

Sample	Retention Time (min)	Peak (% Area)
1. Standard	13.840	74.884
2. WT	13.580	3.854
3. *Mtb-pMV261-rv1357c*	13.792	0.04
4. *Mtb-pMV261-ydeH*	13.835	15.599

Fig. S3. Determination of intracellular cyclic-di-GMP levels in *M. tuberculosis* (WT) and its derivative strains. Chromatograms of (A) standard cyclic di-GMP; (B-D)lysates from various *M. tuberculosis* strains loaded on reverse phase C18 HPLC columns, and eluted with a solvent gradient described under Methods. Cyclic-di-GMP peaks are indicated by red arrows. (E)Peak areas corresponding to the cyclic di-GMP peaks are shown.

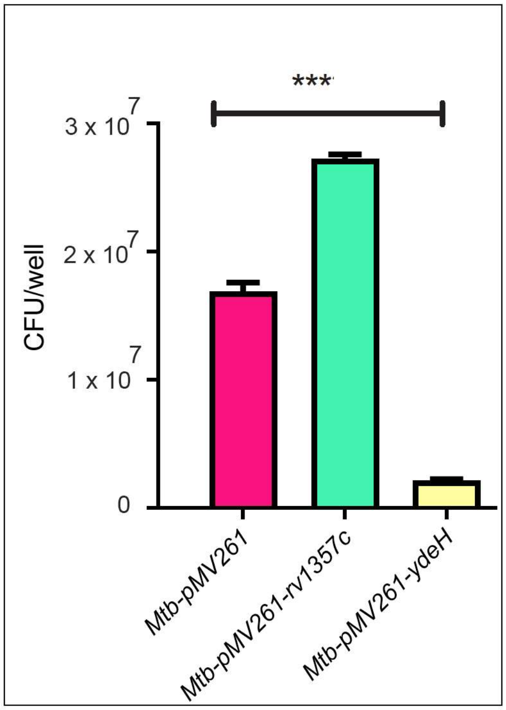

Fig. S4. CFUs of biofilm-derived *M. tuberculosis*. Viability of *M. tuberculosis* strains in biofilms was determined by enumeration of CFUs as described under Methods. Comparisons were made using Kruskal-Wallis followed by Dunn's test; n=9 (including replicates) ***; $P \leq 0.0001$.
